# An efficient single-cell transcriptomics workflow for microbial eukaryotes benchmarked on *Giardia intestinalis* cells

**DOI:** 10.1186/s12864-020-06858-7

**Published:** 2020-06-29

**Authors:** Henning Onsbring, Alexander K. Tice, Brandon T. Barton, Matthew W. Brown, Thijs J. G. Ettema

**Affiliations:** 1 Department of Cell and Molecular Biology, Science for Life Laboratory, Uppsala University, 75123 Uppsala, Sweden; 2 Department of Biological Sciences, Mississippi State University, Starkville, Mississippi State USA; 3 Laboratory of Microbiology, Department of Agrotechnology and Food Sciences, Wageningen University, Wageningen, the Netherlands

**Keywords:** Protists, Microbial eukaryotes, RNAseq, Transcriptomics, Microbial diversity, Smart-seq2, Single cell genomics, *Giardia intestinalis*, Transcriptome, Single-cell RNA sequencing

## Abstract

**Background:**

Most diversity in the eukaryotic tree of life is represented by microbial eukaryotes, which is a polyphyletic group also referred to as protists. Among the protists, currently sequenced genomes and transcriptomes give a biased view of the actual diversity. This biased view is partly caused by the scientific community, which has prioritized certain microbes of biomedical and agricultural importance. Additionally, some protists remain difficult to maintain in cultures, which further influences what has been studied. It is now possible to bypass the time-consuming process of cultivation and directly analyze the gene content of single protist cells. Single-cell genomics was used in the first experiments where individual protists cells were genomically explored. Unfortunately, single-cell genomics for protists is often associated with low genome recovery and the assembly process can be complicated because of repetitive intergenic regions. Sequencing repetitive sequences can be avoided if single-cell transcriptomics is used, which only targets the part of the genome that is transcribed.

**Results:**

In this study we test different modifications of Smart-seq2, a single-cell RNA sequencing protocol originally developed for mammalian cells, to establish a robust and more cost-efficient workflow for protists. The diplomonad *Giardia intestinalis* was used in all experiments and the available genome for this species allowed us to benchmark our results. We could observe increased transcript recovery when freeze-thaw cycles were added as an extra step to the Smart-seq2 protocol. Further we reduced the reaction volume and purified the amplified cDNA with alternative beads to test different cost-reducing changes of Smart-seq2. Neither improved the procedure, and reducing the volumes by half led to significantly fewer genes detected. We also added a 5′ biotin modification to our primers and reduced the concentration of oligo-dT, to potentially reduce generation of artifacts. Except adding freeze-thaw cycles and reducing the volume, no other modifications lead to a significant change in gene detection. Therefore, we suggest adding freeze-thaw cycles to Smart-seq2 when working with protists and further consider our other modification described to improve cost and time-efficiency.

**Conclusions:**

The presented single-cell RNA sequencing workflow represents an efficient method to explore the diversity and cell biology of individual protist cells.

## Background

Protists are undersampled among the eukaryotes in terms of genome and transcriptome sequencing efforts. The scientific community has mainly generated such data for plants, fungi, and animals [1]. Generation of genome and transcriptome data for protists is challenging, since only a small minority of this group have been cultivated under controlled laboratory conditions [2–4]. Methods that are using only a single cell as input can bypass the time-consuming work of establishing a culture. Single-cell genomics is an example of such an approach, which has been applied to expand our knowledge about protist diversity. However, attempts to sequence the genome from single protist cells are often associated with poor genome recovery [5–7]. Another possibility to generate gene content data from uncultivated protists is single-cell RNA sequencing, avoiding the often-problematic, repetitive intergenic regions.

Single-cell RNA sequencing was first tested on protists in a study from 2014 [8] that used the commercial SMARTer kit, achieving a result comparable to conventional sequencing based on RNA extraction from a culture. However, the cells ranged from 50 to 500 μm in size that were analyzed in that study. Single-cell RNA sequencing of a haptophyte and dinoflagellate (8 and 15 μm cell size respectively) were later tested in 2017 by Liu et al. [9], where an updated version of the SMARTer kit (SMART-Seq) was used. In this study only 3% of the transcripts were recovered on average for the haptophyte and 15% for the dinoflagellate. Modifications of the SMART-Seq protocol might be needed to achieve better results for cells that have low RNA content or a durable cell wall. Unfortunately, modifications of the procedure can be complicated when a commercial kit is used, especially since some of the components tend to be kept undisclosed and the kits themselves are expensive per reaction.

In this study we have instead used Smart-seq2 [10] as a starting point, which is fully based on off-the-shelf reagents and performs better than the SMARTer kit, both when it comes to gene detection and coverage [11]. Unlike Liu et al., we have not performed any RNA extraction prior to cDNA synthesis, which could potentially reduce transcript recovery.

The key advantages with Smart-seq2 based workflows are the low price and the fully disclosed components, which makes a protocol easier to modify. However, the disadvantage with relying on off-the-shelf reagents is that getting started can take a long time, and the initial investments can be higher. Therefore, if just a few transcriptomes are going to be generated it could be worth considering commercial kits. We have not compared our protocol to any commercial kit, but we expect that SMART-Seq (Takara) and NEBNext (New England Biolabs) would give satisfying results for many protist lineages as long as the lysis procedure is improved, e.g. with the freeze-thaw cycles suggested in our study. Both SMART-Seq and NEBNext generate full-length cDNA, which is important when working with poorly characterized lineages. There are several microfluidics based solutions for high throughput single-cell RNA sequencing available [12, 13]; these solutions have limited use for protists since they do not generate data for full-length cDNA. Lysis will also be more challenging when microfluidics is used, since freeze-thaw cycles cannot be applied.

In our Smart-seq2 based workflow we have tested different changes, which might improve the generation of cDNA from protists that are difficult to lyse or have a low RNA content. Our modifications of Smart-seq2 offer improved lysis and less dependence on quality control compared to the original protocol. We have benchmarked all protocols tested in this study on *Giardia intestinalis*, for which the genome is sequenced [14]. A key problem limiting the accessibility of RNAs is the lysis of the protist cells. Also for cells with low RNA content, there can be a problem with unspecific amplification due to changed balance between the concentration of oligos and mRNA of the cell [15]. The potential problem with lysis is addressed by using freeze-thaw cycles in − 80 °C chilled isopropanol, which previously have been reported as a successful lysis procedure [16, 17]. Besides the improved lysis we already know can be crucial, we test modifications of Smart-seq2 to maximise cost-efficiency and minimise artifacts during cDNA synthesis.

## Results

### Gene detection and coverage

**Fig. 1:**
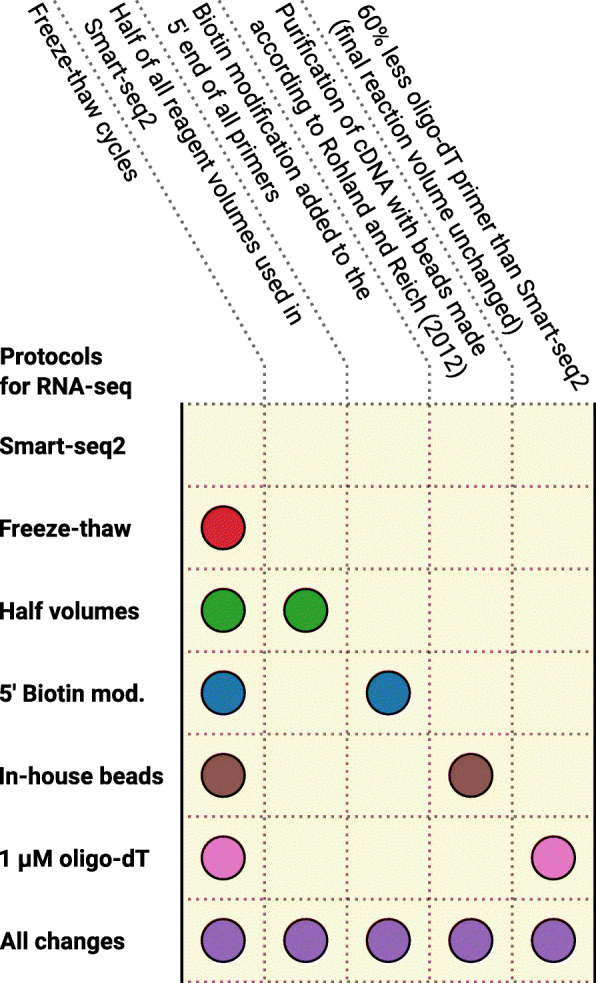
Overview of how the protocols tested in this study differ from Smart-seq2

Single *G. intestinalis* trophozoites were sorted using fluorescence-activated cell sorting and seven different protocols for generation of transcriptomes were applied, including Smart-seq2 and modified versions of Smart-seq2 (Fig. 1). Freeze-thaw cycles were added to all six modifications of Smart-seq2. Additionally, five of the modified versions of Smart-seq2 had one or all of the following changes: biotinylated 5′ end of primers, other beads for cDNA purification, lower reaction volume and less oligo-dT primers than Smart-seq2 (see methods for details).

**Fig. 2:**
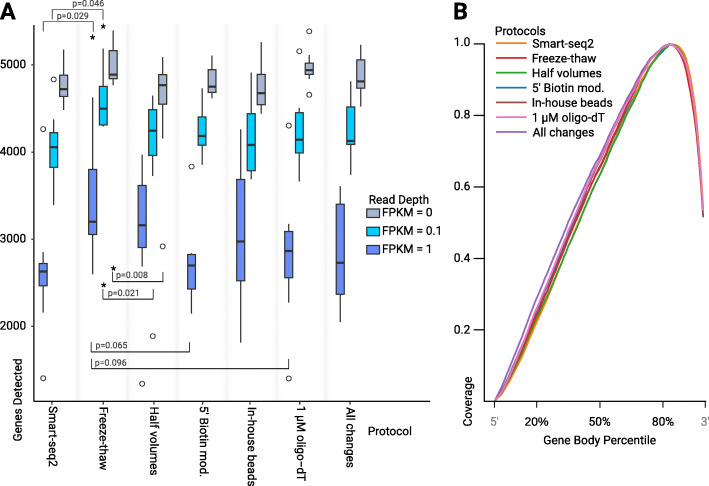
Transcriptome quality statistics. **a** Box and whisker plot showing number of genes detected for all permutations at three expression levels. An asterix indicates significance when compared to our “freeze-thaw” protocol (*p* < 0.05). *P*-values below plots indicate that the performance of the treatment was worse at gene detection. **b** Average gene-body coverage for all genes detected in the *G. intestinalis* genome by each protocol

The sequencing data generated from all transcriptomes corresponded to 703 Gbp, covering 55 individual cells. We detected on average 4524 to 4992 genes in all tested protocols (Fig. 2a), representing 70–77% of the total protein coding genes in the genome of *G. intestinalis* [14]. Using fragments per kilobase of transcript per million mapped reads (FPKM) allowed us to take the abundance of transcripts into consideration in our analysis. All protocols, except the version where all tested changes are implemented, differ by only one treatment compared to Smart-seq2 with freeze-thaw cycles. Therefore, we used this “Freeze-thaw” protocol as the point of reference in our pairwise comparisons. Using the unmodified Smart-seq2 lead to significantly fewer genes being detected among the medium and high abundance transcripts (FPKM > 0.1 and > 1) than when the “Freeze-thaw” protocol was applied. When half volumes of the standard reagents were used throughout the protocol, significantly fewer genes were detected for both low and medium abundance transcripts (FPKM > 0 and > 0.1) compared to the “Freeze-thaw” protocol.

We also tested the use of biotinylated primers, reduced concentration of oligo-dT primers, beads made in-house or a combination of all modifications of Smart-seq2 tested in this study, neither of these protocols performed significantly different from the “Freeze-thaw” protocol. However, we saw a marginal decreases in gene detection when using 1 μM oligo-dT (generalized linear model, *p* = 0.096), and biotinylated primers (generalized linear model, *p* = 0.065) at a read depth of FPKM > 1 (see Table 1). Unmodified Smart-seq2, as well as all our modified protocols, show a 3´ bias in gene-body coverage (Fig. 2b). This bias is common to protocols that use oligo-dT priming during cDNA synthesis [18].

**Table 1:** Generalized linear model comparing gene detection of the Freeze-thaw protocol to the other tested protocols. Comparison of the number of genes detected for Smart-seq2, and five modified Smart-seq2 variants, against our “Freeze-thaw” protocol using a generalized linear model with a negative binomial error distribution. Significant *p*-values (<= 0.05) are indicated with an asterisk

FPKM	Protocol	Estimate	StdError	z-value	Pr(>|z|)
0	Smart-seq2	−0.045146	0.036829	−1.226	0.22026
0	Half volumes	−0.098322	0.036848	−2.668	0.00762*
0	5′ Biotin mod.	−0.035167	0.038118	−0.923	0.35622
0	In-house beads	−0.050162	0.03683	−1.362	0.17321
0	1 μM oligo-dT	−0,004165	0.036814	−0.113	0.90992
0	All changes	−0.022638	0.036821	−0.615	0.53867
0.1	Smart-seq2	−0.12486	0.06261	−1.994	0.0461*
0.1	Half volumes	−0.14494	0.06261	−2.315	0.0206*
0.1	5′ Biotin mod.	−0.07958	0.0648	−1.228	0.2194
0.1	In-house beads	−0.10104	0.0626	−1.614	0.1065
0.1	1 μM oligo-dT	−0.07918	0.0626	− 1.265	0.2059
0.1	All changes	−0.0756	0.0626	−1.208	0.2271
1	Smart-seq2	−0.26975	0.12333	−2.187	0.0287*
1	Half volumes	−0.11903	0.12331	−0.965	0.3344
1	5′ Biotin mod.	−0.23547	0.12766	−1.845	0.0651
1	In-house beads	−0.13111	0.12331	−1.063	0.2876
1	1 μM oligo-dT	−0.20511	0.12332	−1.663	0.0963
1	All changes	−0.20074	0.12332	−1.628	0.1036

### Identification of phylogenetic markers

**Fig. 3:**
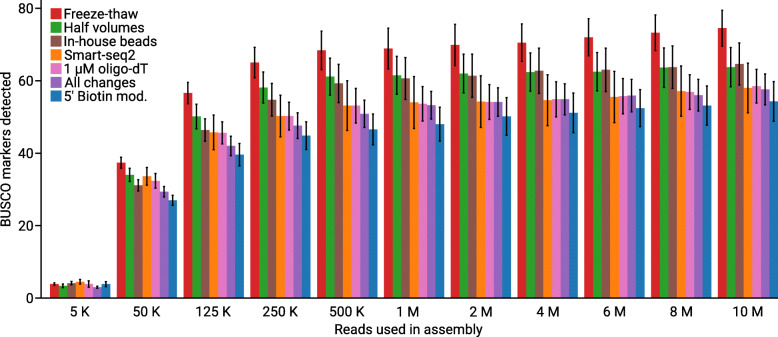
Number of BUSCO markers found in de novo assemblies based on different amounts of data. Bars represent the average number of BUSCO markers found in the de novo assemblies, error bars represent standard error. The average number of BUSCO markers found varied between 54 to 75 (out of the 303 proteins in the eukaryota_odb9 dataset). Retrieving around 54 to 75 markers from our single-cell transcriptomes can be compared to the reference transcriptome of *G. intestinalis* on NCBI, which encodes 146 of the BUSCO markers from eukaryota_odb9

To obtain a rough estimate how much data is needed to be able to extract marker genes to build a multi-gene concatenated alignment for phylogenomics of an unknown protist, we down-sampled our data and ran multiple de novo assemblies in several iterations (Fig. 3). Generally among the comparisons, based on different number of reads used in the assembly, we observe that Smart-seq2 with freeze-thaw cycles identified more markers than Smart-seq2, 1 μM oligo-dT, 5′ biotin modification and when all changes where applied. As a proxy for a phylogenomic analysis dataset, we calculated the number of observed BUSCO from the Eukaryota odb9 dataset. The number of BUSCO markers detected did not increase much if more sequencing data was generated beyond 500 thousand read pairs, which correspond to 150 Mbp sequencing data. This indicates that a low amount of data is needed if the only goal is to find markers for a phylogenomic analysis. We could find 5 bacterial BUSCO markers that caused an insignificant overestimation of the transcript recovery, indicating contamination is not affecting our conclusions.

## Discussion

By performing Smart-seq2, and six alternative modifications of this protocol, we generated 55 transcriptomes of single *G. intestinalis* cells. The raw sequencing reads allowed us to generate statistics for gene detection and gene-body coverage by mapping to the *G. intestinalis* genome [14]. Our experiment shows that adding six freeze-thaw cycles to the Smart-seq2 protocol will not decrease the RNA quality in a way that negatively affects gene detection or gene-body coverage. Adding these freeze-thaw cycles actually turned out to significantly increase the number of genes detected among the two highest read depths analyzed. Because of this improvement and since we expect that many protists are harder to lyse than the mammalian cells used to optimized Smart-seq2, we suggest that freeze-thaw cycles should be used when generating protist transcriptomes from single-cell input. Because our experience is that the freeze-thaw cycles can be necessary to get a successful cDNA library [16], we have used freeze-thaw in all of our modifications of the Smart-seq2 protocol. Therefore, Smart-seq2 with freeze-thaw cycles becomes the point of reference and will be used as our control in pairwise comparisons to other tested protocols.

The only modified version of Smart-seq2 we tested in this study, which lead to significantly fewer genes detected, was when we reduced all reagent volumes to half of what is used in the original protocol. The lower performance could be due to the unfavorable change in ratio between reaction volume and surface area of the test tube wall, which can absorb nucleic acids [19]. Despite the lower performance, reducing all volumes by half may be considered in experimental design due to cost savings associated with using less reagents, which could be important when running many reactions.

It has been reported that modifications of Smart-seq2 are necessary when working with cells with extremely low RNA content, e.g. concatamerization of the template switching oligo can prevent the generation of usable cDNA libraries (Picelli 2016). To prevent such generation of background during cDNA synthesis we tried adding a 5′ biotin modification for all primers, which is also recommended in an updated version of the Smart-seq2 [20]. Adding the 5′ biotin modification did not increase the number of genes detected and the number of BUSCO markers were fewer than what was recovered from the control. At the same time when the biotin modification was not used, the concatamers [21] that would be visible as a ‘hedgehog’ pattern around 100 to 1000 bp in the fragment length analysis, were never observed (see Additional file 1). Based on recommendations from other studies this option can be considered as an insurance against failed cDNA generation, especially for cells with lower RNA content than *G. intestinalis*.

Another protocol modification that could reduce the amount of artifacts is changing the concentration of primers, which we tested by decreasing the concentration of oligo-dT by 60%. This was done since the imbalance of primers and mRNA has been claimed to be one of the reasons why background is generated when working with cells that have low mRNA content [15]. Reducing the concentration of oligo-dT with 60% did not increase the number of genes detected, and fewer BUSCO markers were found. Therefore, using less oligo-dT should not be considered for cells with as much RNA as *G. intestinalis* or more, if the goal is to maximize transcript recovery.

**Fig. 4:**
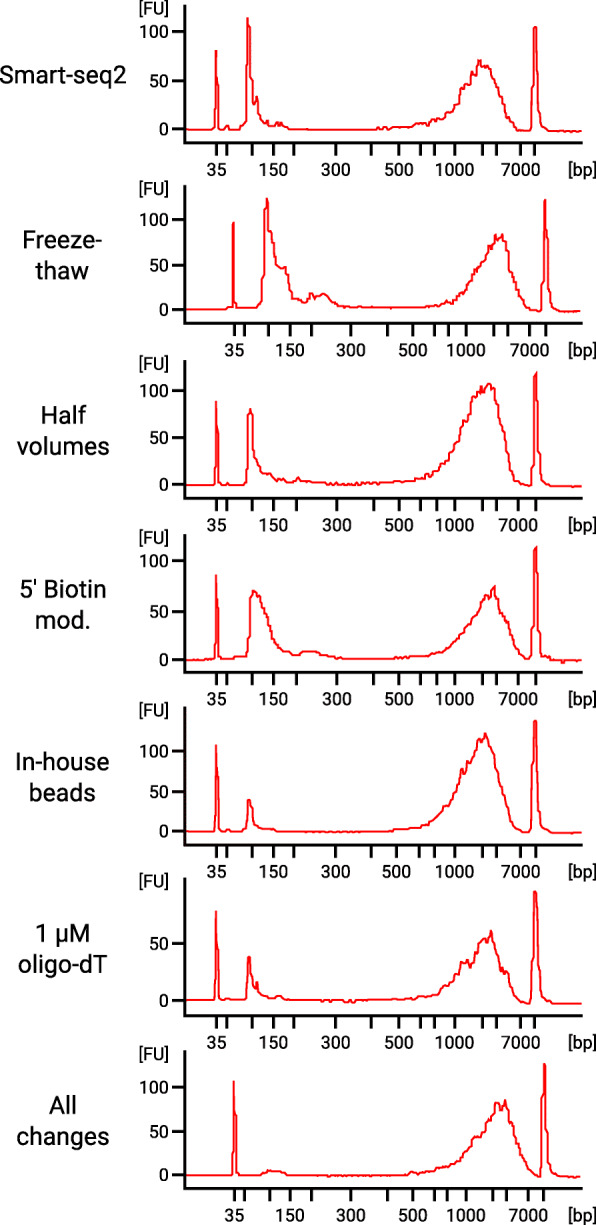
Fragment length analysis of seven cDNA libraries covering each variation of the protocols tested

Besides the previously discussed oligo-concatamers, an artifact that we did see in our fragment length analysis was the formation of primer dimers. We could reduce the amount of primer dimers by preparing beads for purification of the amplified cDNA (Fig. 4). However, we did not observe any aspect of the protocol that improved by this change, except lower cost of consumables for DNA purification compared to Smart-seq2.

If a high number of transcriptomes are going to be generated, we recommend using all modifications of Smart-seq2 tested in this study. However, the “All changes” protocol did not lead to higher transcript recovery compared to the control. The important benefit of the “All changes” workflow is that the user becomes less dependent on the time-consuming and costly fragment length analysis step. When generating many transcriptomes it is advantageous to be able to identify failed reactions by just measuring the DNA concentration. If all modifications tested in this study are applied all at once, then the failed reactions will typically measure well below the lowest recommended input for sequencing library preparation. Therefore this will save time and money by reducing the need for fragment length analysis, while also less reagents and cheaper purification beads are used. However, checking the fragment length distribution on a subset of the generated cDNA libraries is always recommended. Fragment length analysis allows detection of ribonuclease contamination and can prevent the user from proceeding to the next step in the workflow with a degraded sample. If there is no equipment available for detailed fragment length analysis, or if the user wants to reduce cost, additional amplification of the sequencing libraries combined with gel electrophoresis has previously been used as an alternative [22].

## Conclusions

All variations of the RNA sequencing workflow tested in this study were only benchmarked on *G. intestinalis*. Each variation of the Smart-seq2 could be more or less beneficial with other species, where RNA content should be an important factor. The protocols suggested here may serve as a starting point for other protists.

Our results from testing seven different protocols for generation of cDNA suggests that freeze-thaw cycles should be added to a single-cell transcriptomics workflow for protists. To save money, all volumes in Smart-seq2 can be reduced to half and lab-prepared purification beads can be used, but neither of these changes leads to any improvements in gene detection. Actually, using half of the recommended Smart-seq2 volumes might reduce the transcript recovery. A 5′ biotin modification of the primers can be considered as an insurance against concatamers, but this change could be at the expense of lower transcript recovery as well.

To become less dependent on quality control, all changes tested in this study can simultaneously be applied in one protocol. The dependency on quality control is reduced since failed reactions will have a cDNA concentration close to 0, and therefore it is possible to discard unsuccessful cDNA libraries only based on DNA concentration.

Transcriptomes encoding markers for multi-gene concatenated phylogenies can be generated with single-cell RNA sequencing, even with low amount of sequencing data. All variations of Smart-seq2 tested in this study are suitable options for generation of data to perform phylogenomic analysis. Therefore, instead of optimizing transcript recovery, factors such as time or cost-efficiency can be considered.

## Methods

### Cell sorting

Trophozoites of *Giardia intestinalis* (strain ATCC 50803, WB clone C6) were grown to confluence in 10 mL flat bottom tubes (NUNC) and detached on ice for 10 min. The cell suspension was transferred to a 15 mL Falcon tube and centrifuged at 500 *g* for 10 min. The supernatant was discarded and resuspended in 500 μL 1xPBS. Prior to sorting, the sample was prepared using a cell suspension of harvested trophozoites diluted 10 times in sterile filtered 1xPBS and stained with DAPI and Propidium Iodide (PI) to a final concentration of 1 μg/mL and 200 nM respectively for 10 min. The sorting was performed with a MoFlo Astrios EQ (Beckman Coulter, USA) flow cytometer using the 355 and 532 nm lasers for excitation, a 100 μm nozzle, sheath pressure of 25 psi and 0.2 μm filtered 1xPBS as sheath fluid. Live cells were identified using scatter properties in combination with a singlets gate and exclusion of dead PI positive cells. Individual cells were deposited into 12 × 8-well strips containing 2.3 μl or 4.3 μl of lysis buffer using a CyCloneTM robotic arm and the most stringent single cell sort settings (e.g single mode, 0.5 drop envelope).

The lysis buffer were for some reactions prepared according to Smart-seq2 [10], and altered in some of the modified versions of the protocol (see the methods paragraph “cDNA synthesis” for details). A UV-laser (355 nm) was used for excitation of DAPI and emission was collected by a 448/59 nm filter. Excitation of PI and collection of emitted light was done with a 532 nm laser with a 622/22 nm filter. Side scatter was used as trigger channel. The plate and sample holder were kept at 4 °C during the sort. The 8-strips were sorted two by two, quickly spun down and temporarily stored at − 20 °C until the sort was finished before transfer to a − 80 °C freezer.

### cDNA synthesis

The cDNA was prepared according to Smart-seq2 [10], and six modified versions of Smart-seq2, using 24 cycles of cDNA amplification in each case. Our experience is that increasing the amplification cycles to 24 is a conservative choice that will allow the generation of enough cDNA for library preparation, even for cells with low mRNA content. We generated 8 cDNA libraries for every version of the protocol. All six modified versions of Smart-seq2 included freeze-thaw cycles as an extra lysis step. The freeze-thaw cycles were performed by first thawing the frozen cells in room-tempered water for 10 s directly after taken out of the freezer. Immediately after the 10 s thaw, the tubes were frozen down again in − 80 °C isopropanol for 10 s. This freeze-thaw cycle was repeated six times.

The specific changes applied for each of the six protocols were 1) No additional changes to Smart-seq2 besides the freeze-thaw cycles. 2) Decreasing the oligo-dT primer concentration to 1 μM, instead of 2.5 μM, in the first mix of primer, dNTP and lysis that is added to the cell. 3) Using the beads described by N. Rohland and D. Reich [23], with a 17% PEG concentration, for purification of the amplified cDNA. 4) All volumes were reduced to half of what is used in the original Smart-seq2 protocol. 5) Adding a 5′ biotin modification to all primers, including the one used for template switching. 6) Using all these changes in combination, including freeze-thaw cycles, decreased oligo-dT concentration, using the beads made in-house, reducing all volumes by half and 5′ biotin modification added to primers (see Additional file 3 for details). Negative controls where done by excluding the FACS step, generating tubes without cells. Four replicates of negative controls for the “In-house beads” protocol were generated.

### Tagmentation and sequencing

DNA concentration was measured with Qubit dsDNA HS Assay Kit (Thermo Fisher Scientific). Fragment length analysis was done using Agilent High Sensitivity DNA Kit with a 2100 Bioanalyzer Instrument on a subset of the purified cDNA (see Additional file 1). The purified cDNA was then diluted so each sequencing library preparation reaction had a 1.3 ng input of DNA, followed by using the Nextera XT DNA Library Preparation Kit (Illumina). In our workflow we could produce sequencing libraries of good quality with a DNA input of 1 ng up to 1.6 ng, therefore we used and input in the middle of this interval. One Nextera XT library failed, leading to that only 7 replicates based on the protocol using 5′ biotinylated primers were included in the sequencing run. A total of 55 single-cell transcriptomes were sequenced on a separate lane of Illumina NovaSeq S4 (2 × 150 bp reads). No negative controls were sequenced since the cDNA concentration was substantially lower when a cell was excluded compared to the reactions in which a cell was included. Sequencing data from the negative controls could have been useful to estimate cross-contamination, but our experimental design does not support the detection of such contaminants.

### Read mapping and quantification

Sequencing data quality was assessed using FastQC v0.11.8 [24] and visualized using MultiQC [25]. Low quality bases and adaptors were removed using Trimmomatic v0.39 with the options “ILLUMINACLIP: 2:30:10 LEADING:5 TRAILING:5 SLIDINGWINDOW:5:16 MINLEN:60” and the NexteraPE-PE.fa to which we manually added primer sequences used in Smart-seq2 to be removed TSO (5´- AAGCAGTGGTATCAACGCAGAGTACATGGG-3´), oligo-dT (5´- AAGCAGTGGTATCAACGCAGAGTACTTTTTTTTTTTTTTTTTTTTTTTTTTTTTT-3´), and ISPCR (5´- AAGCAGTGGTATCAACGCAGAGT-3´) [26]. Reads were then mapped to the *G. intestinalis* genome (GCF_000002435.1) using TopHat2 with default settings [27]. TopHat2 is splice-aware, but does not perform as well as more recently developed software such as HISAT2, since only eight spliceosomal introns has been found in the genome of *G. intestinalis* [28]. Additional file 2 visualizes an example of our mapping results from TopHat2 for a 30 kb region of the *G. intestinalis* genome (contig NW_002477110.1) selected randomly using the “random” function of bedtools v. 2.29 [29] with the options -l 30,000 -n 1 and displayed using the Broad Institute’s Integrative Genomics Viewer v. 2.7.2 [30]. The mapped reads are derived from one “All changes” library (GenBank accession: SRR9222552) selected at random from a directory containing all libraries using the linux/python command “ls -1 | python -c “import sys; import random; print (random.choice(sys.stdin.readlines()).rstrip())””. The python scripts geneBody_coverage.py and FPKM_count.py from RSeQC-2.6.4 were used to examine read distribution across genes and calculate FPKM values for all libraries respectively [31]. The box and whisker plot for number of genes detected was generated in R using the ggplot package. While the line graph showing gene-body coverage was made using matplotlib via a custom python script, which is publicly available on github (https://github.com/atice/Code-Used-in-Onbring-et-al/blob/master/Gene_Body_Coverage_plotmaker.py).

### Statistical analyses

We compared the number of genes detected at three expression/abundance levels (FPKM > 0, > 0.1, > 1) for unmodified Smart-seq2 and five protocol variants against our “Freeze-thaw” protocol. We used a generalized linear model with a negative binomial error distribution to correct for overdispersion. All statistical analyses were conducted with the *glm* module in R.

### BUSCO analysis

Separate assemblies were done for each cell using Trinity v2.4.0 [32]. For every cell we assembled 11 different assemblies using the following number of reads as input: 10 million, 8 million, 6 million, 4 million, 2 million, 1 million, 500 thousand, 250 thousand, 125 thousand, 50 thousand, 5 thousand. Each assembly was then analyzed with BUSCO v3.1.0 [33], using the eukaryota_odb9 dataset. A BUSCO analysis was also done on the reference transcriptome of *G. intestinalis* from the NCBI database. DIAMOND v0.9.24.125 [34] in blastx mode, with the --more-sensitive setting, was used to assess if contamination had an effect on the BUSCO analysis by querying the BUSCO hits against NCBI *nr* database. The DIAMOND blastx search was restricted to human, fungi and bacteria by using the --taxonlist setting.

## Supplementary information

**Additional file 1.** Fragment length distribution for all cDNA libraries checked with Agilent High Sensitivity DNA Kit.

**Additional file 2. **TopHat2 mapping of Illumina sequencing reads to the *G. intestinalis* genome. Integrative Genomics Viewer display of read mapping results from “All Changes” replicate SRR9222552 using TopHat2 to a randomly selected 30 kb region of the *G. intestinalis* genome (contig NW_002477110.1).

**Additional file 3.** How to synthesize and amplify cDNA described in a protocol format.

## Data Availability

All raw sequencing reads generated in this study have been submitted to NCBI under the BioProject PRJNA545787, which includes the “All changes” library SRR9222552 used to visualize our mapping results. The *G. intestinalis* genome used as reference can be accessed on NCBI under the BioProject PRJNA15590. The python script used to generate the figure showing gene-body coverage is publicly available on github (https://github.com/atice/Code-Used-in-Onbring-et-al/blob/master/Gene_Body_Coverage_plotmaker.py). The eukaryota_odb9 dataset used to assess transcript recovery is an integrated part of the BUSCO package (available on https://busco.ezlab.org).

## Abbreviations

Gbp: Giga base pairs
bp: Base pair
BUSCO: Benchmarking universal single-copy orthologs
DAPI: 4′,6-diamidino-2-phenylindole
FACS: Fluorescence activated cell sorting
FPKM: Fragments per kilobase of transcript per million mapped reads
NCBI: National Center for Biotechnology Information
*nr*: Non-redundant
PBS: Phosphate buffered saline
PI: Propidium iodide
