# Supplementary figures and images for: An efficient single-cell transcriptomics workflow for microbial eukaryotes benchmarked on Giardia intestinalis cells
Source: BMC Genomics. 2020 Jun 29;21:448. doi: 10.1186/s12864-020-06858-7 (PMC7325058; doi:10.1186/s12864-020-06858-7)

### Smart-seq2

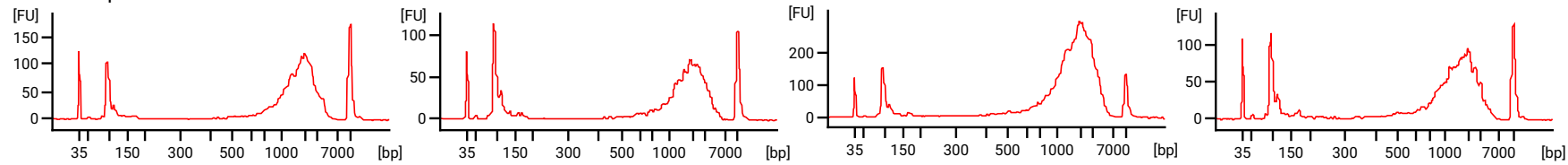

### Freeze thaw

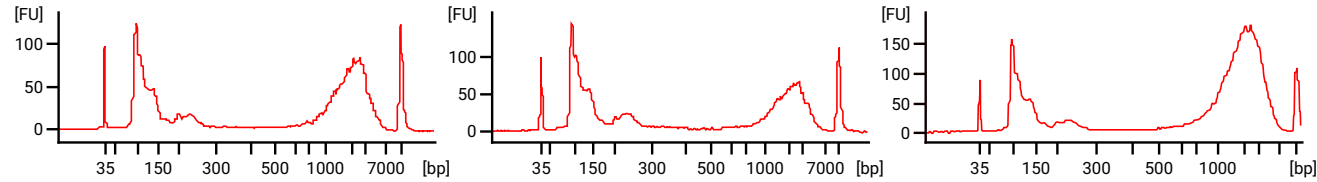

### All changes

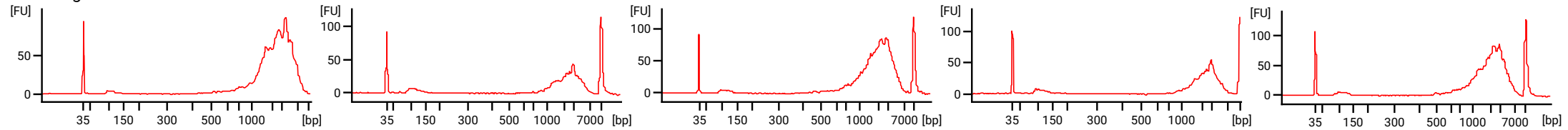

### In-house beads

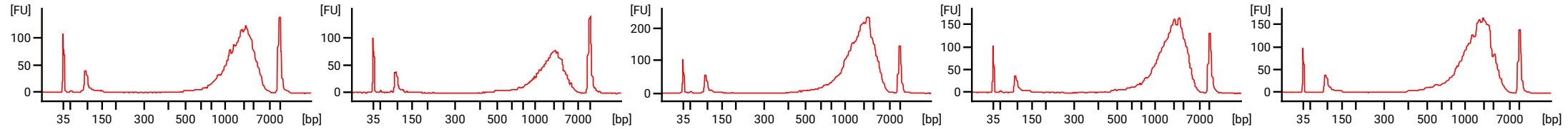

### Half volumes

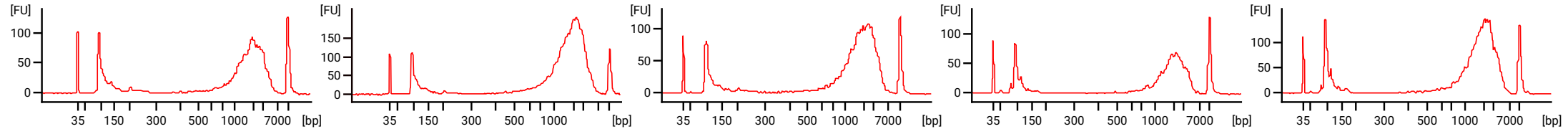

### 5' Biotin mod.

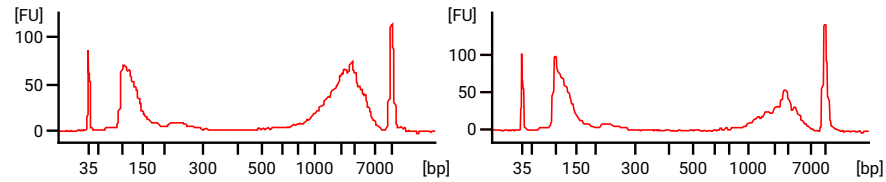

### 1 $\mu$ M oligo-dT

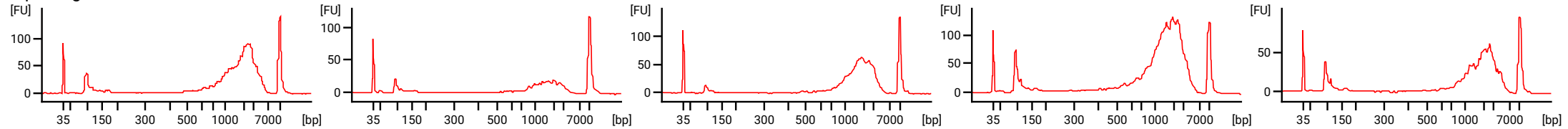

Supplement: Supplementary file 1 — Additional file 1. Fragment length distribution for all cDNA libraries checked with Agilent High Sensitivity DNA Kit. [file 12864_2020_6858_MOESM1_ESM.pdf]

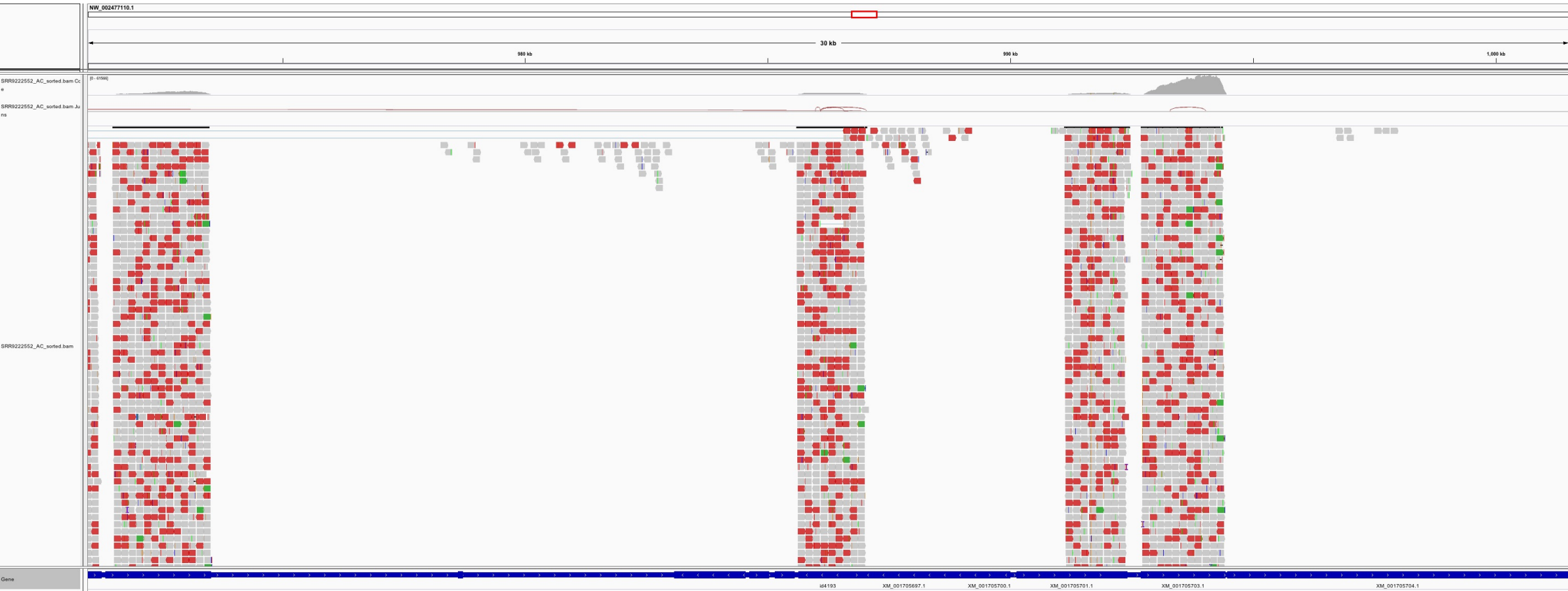

Supplement: Supplementary file 2 — Additional file 2. TopHat2 mapping of Illumina sequencing reads to the G. intestinalis genome. Integrative Genomics Viewer display of read mapping results from “All Changes” replicate SRR9222552 using TopHat2 to a randomly selected 30 kb region of the G. intestinalis genome (contig NW_002477110.1). [file 12864_2020_6858_MOESM2_ESM.pdf]
