# Supplementary material for: An efficient single-cell transcriptomics workflow for microbial eukaryotes benchmarked on Giardia intestinalis cells
Source: BMC Genomics. 2020 Jun 29;21:448. doi: 10.1186/s12864-020-06858-7 (PMC7325058; doi:10.1186/s12864-020-06858-7)
Supplement: Supplementary file 3 — Additional file 3. How to synthesize and amplify cDNA described in a protocol format. [file 12864_2020_6858_MOESM3_ESM.pdf]

Supplemental information

An efficient single-cell transcriptomics workflow for microbial eukaryotes benchmarked on *Giardia intestinalis* cells

Henning Onsbring[1,a], Alexander K. Tice[2,a], Brandon T. Barton[2], Matthew W. Brown[2,b] and Thijs J. G. Ettema[1,b,c]

[1] Department of Cell and Molecular Biology, Science for Life Laboratory, Uppsala University, 75123 Uppsala, Sweden
[2] Department of Biological Sciences, Mississippi State University, Mississippi State, MS

Introduction
The consumables needed for generation of cDNA libraries from single protist cells are described in Table 1. Experimental procedures should be done in a PCR workstation equipped with a UV lamp before the cDNA amplification step.

Table 1. Consumables needed for cDNA generation from single-cell input.

Consumable	Manufacturer	Catalog number
RNaseZap	Ambion	AM9780
DNA AWAY	Thermo Scientific	7010
Magnesium chloride hexahydrate	Sigma-Aldrich	M2393
UltraPure DNase/RNase-Free Distilled Water	GIBCO	10977
Triton X-100	Sigma-Aldrich	T9284
SuperScript II Reverse Transcriptase kit	Invitrogen	18064-014
Betaine	Sigma-Aldrich	61962
Recombinant RNase inhibitor	Clontech	2313A
dNTP Set 100 mM Solutions	Thermo Scientific	R0181
KAPA HiFi HotStart ReadyMix	Roche	KK2601

Oligo nucleotides
Primer Oligo-dT30VN (biomers.net):
/biotin/AAGCAGTGGTATCAACGCAGAGTACTTTTTTTTTTTTTTTTTTTTTTTTTTTTTTVN
(V = A, C or G, N = A, C, T or G)

Primer TSO (Exiqon):
/biotin/AAGCAGTGGTATCAACGCAGAGTACATrGrG+G
(r = RNA base, + = LNA base)

Primer ISPCR (biomers.net):
/biotin/AAGCAGTGGTATCAACGCAGAGT

Protocol for cDNA generation and amplification from single protist cells

Adapted from Smart-seq2

1. Clean the hood with RNaseZap and DNA AWAY. Bring as much pipette tips, water and empty tubes that is needed into the hood. UV irradiate hood for at least 30 min.

2. To handle low volumes of $MgCl_2$, 1.2 µl of 1 M $MgCl_2$, can be added to 5.8 µl of water and from this mix the total needed volume of the both reagents can be transferred to the RT mix. Prepare the lysis mix and part of the reverse transcriptase mix by following the steps:

   a) Mix 49 µl H_2O with 1 µl Triton X-100, vortex and spin down.
   b) Mix 35.5 µl H_2O with 9.5 µl of the 2% (vol/vol) Triton X-100.
   c) Mix the first-strand buffer, DTT, betaine, water and $MgCl_2$ from the RT mix in Table 2.
   d) Add 1 µl RNase inhibitor to the tube with 9 µl of 0.2% (vol/vol) Triton X-100 solution.
   e) Add 0.5 µl lysis mix for each reaction needed plus one to a new tube.
   f) Add 0.2 µl 25 mM dNTP mix for each reaction needed plus one to the lysis mix.
   g) Add 0.2 µl oligo-dT primer for each reaction needed plus one to the lysis mix.
   h) Bring TSO, positive control into the clean room and put on ice. Add TSO when thawed.

3. Start the thermal cycler with a 72 ^oC incubation temperature.

4. Right after taking the 0.2 ml tubes containing a cell in 1.4 µl water out of -80 ^oC, perform an incubation step of 10 s in room temperature water followed by 10 s in -80 ^oC chilled isopropanol. Repeat this freeze-thaw cycle five times and bring the tubes in a benchtop cooler tube rack to the hood.

5. Add 0.9 µl of the lysis mix to each tube.

6. Gently vortex and spin down the cells with lysis mix when the tube content has thawed, followed by 72 ^oC incubation for 3 min. Immediately put the tubes back **on ice** after the incubation.

7. Briefly spin down the samples so the content are collected at the bottom of the tubes. After the spin, put the tubes immediately back **on ice**.

8. Prepare the remaining part of the RT mix for all reactions plus one additional reaction by combining and mixing the reagents listed in Table 2.

Table 2. RT mix, volumes for a single reaction.

**Component**	**Volume (µl)**	**Final concentration**
Superscript II first-strand buffer (5×)	1.00	1x
Betaine (5 M)	1.00	1 M
DTT (100 mM)	0.25	5 mM
Nuclease-free water	0.145	-
$MgCl_2$ (1 M)	0.03	6 mM
TSO (100 µM)	0.05	1 µM
RNase inhibitor (40 U μl^{-1})	0.125	1 U μl^{-1}
SuperScript II reverse transcriptase (200 U μl^{-1})	0.25	10 U μl^{-1}
Total volume	2.85	-

9. Add 2.85 µl of the RT mix to the samples from step 7. Mix the reactions by carefully vortexing the samples followed by a quick spin-down.

10. Incubate the reactions according to Table 3, with the lid temperature set to 105 ^{o}C.

Table 3. Reaction temperatures during reverse transcription.

**Cycle**	**Temperature (^{o}C)**	**Time (min)**
1	42	90
2-11	50	2
	42	2
12	70	15
13	4	∞

11. Prepare the PCR mix needed for amplification of the first-strand reaction for all samples plus one additional reaction according to Table 4.

Table 4. PCR mix, volumes for a single reaction.

**Component**	**Volume (µl)**	**Final concentration**
First-strand reaction	5	-
KAPA HiFi HotStart ReadyMix (2x)	6.25	1x
IS PCR primers (10 µM)	0.125	0.1 µM
Nuclease-free water	1.125	-
Total volume	12.5	-

12. Add 7.5 µl of PCR mix to each tube incubated in step 10. Vortex the tubes to mix, and spin down the liquid to the bottom of the tubes.

13. Perform the PCR in a thermal cycler, with the lid temperature set to 105 ^{o}C, by using the program in Table 5. A PCR program with 18 cycles can probably be used for protists around 50 µm cell size. Usually 24 cycles are enough for smaller cells with cell sizes around 5-10 µm.

Table 5. PCR program with 18 cycles.

**Cycle**	**Denature**	**Anneal**	**Extend**	**Hold**
1	98 ^{o}C, 3 min	-	-	-
2-19	98 ^{o}C, 20 s	67 ^{o}C, 15 s	72 ^{o}C, 6 min	-
20	-	-	72 ^{o}C, 6 min	-
21	-	-	-	4 ^{o}C

14. After step 13 is performed, the PCR product should be purified and then the preferred library preparation protocol can be used to generate sequencing libraries.

Cut-off for DNA purification

Smart-seq2 uses Agencourt Ampure XP beads (1:1, beads:cDNA), an alternative cut-off is shown in Figure 1 that removes more primer-dimer than the original Smart-seq2 cDNA purification procedure.

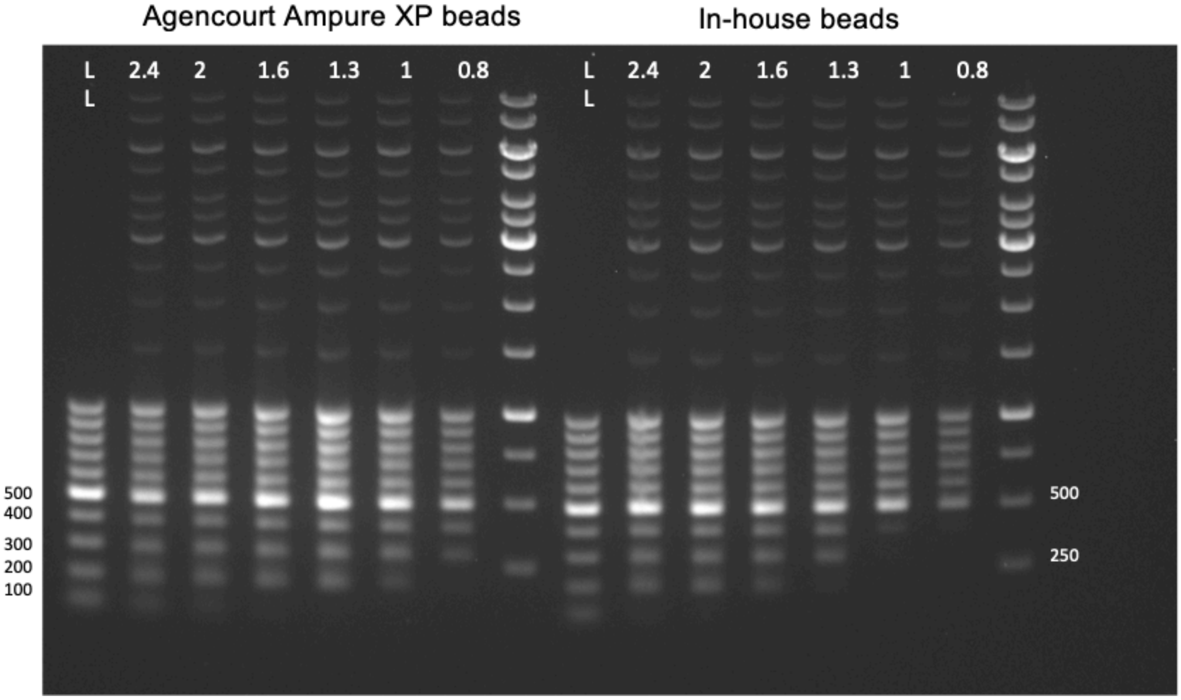

Figure 1. DNA ladder purified with Ampure and In-house beads (0.8:1 – 2.4:1, beads:DNA).
